# Schulbezogene Einstellungen von Kindern aus bildungsfernen Milieus in der Corona-Pandemie

**DOI:** 10.1007/s35834-022-00333-x

**Published:** 2022-05-04

**Authors:** Britta Klopsch, Carsten Rohlfs

**Affiliations:** 1 Institut für Schulpädagogik und Didaktik, Karlsruher Institut für Technologie (KIT), Karlsruhe, Deutschland; 2 Institut für Erziehungswissenschaft, Pädagogische Hochschule Heidelberg, Heidelberg, Deutschland

**Keywords:** Homeschooling, Lockdown, Soziale Benachteiligung, Bildungsferne Milieus, Einstellungen, Lernpotenziale, Homeschooling, Lockdown, Social disadvantage, Educationally disadvantaged milieus, Attitudes, Learning potential

## Abstract

Das Distanzlernen im Lockdown benachteiligt, darin besteht weitgehend Konsens, insbesondere Kinder und Jugendliche aus bildungsfernen Milieus. Die Schere zwischen Privilegierten und Zurückgelassenen, die in Deutschland ohnehin stark geöffnet ist, weitet sich durch die COVID-19-Pandemie immer deutlicher. Empirische Belege für diesen pandemiebedingten Wirkzusammenhang sind allerdings noch wenig belastbar, und selten werden in den entsprechenden Untersuchungen Schüler:innen selbst befragt und noch weniger solche, die an Schulen in herausfordernder Lage unterrichtet werden. Vorliegender Beitrag referiert eine quantitative Studie an Grundschulen in besonders benachteiligter Umgebung während des Lockdowns im Frühjahr 2021 und fragt nach individuellen Einstellungen, Haltungen und Lernerfahrungen der Schüler:innen während des Homeschoolings. Ziel war es, nicht pauschalisierend eine Gruppe von Heranwachsenden als benachteiligt und damit als abgehängt zu definieren, sondern empirisch zu untersuchen, welche individuellen Faktoren, Rahmenbedingungen und Wirkmechanismen dazu führen, dass einige dieser Schüler:innen das Lernen im Lockdown erfolgreicher gestalten können als andere mit zunächst gleichen Ausgangsbedingungen. Die Datenanalysen ergaben vier stabile Cluster, die auf unterschiedliche Ausprägungen der Student Agency aufbauen und Potenziale und Anknüpfungsmöglichkeiten aufzeigen, um Lernende aus bildungsfernen Milieus individuell zu fördern – im Homeschooling und im Präsenzunterricht.

## Abstract (en)

There is widespread consensus that distance learning in lockdown discriminates children and young people from educationally disadvantaged backgrounds in particular. The gap between the privileged and the left-behind, which is already wide open in Germany, is widening more and more as a result of the COVID 19 pandemic. Empirical evidence for this pandemic-related effect is, however, still scarce, and the corresponding studies rarely include students themselves, and even less so those who are taught at schools in challenging situations. This paper reports on a quantitative study of elementary schools in particularly disadvantaged settings during the lockdown in spring 2021. It asks about individual attitudes and learning experiences of students during homeschooling. The goal was not to define a group of adolescents as disadvantaged and thus as disconnected, but to empirically investigate which individual factors, framework conditions, and mechanisms of action lead to some of these students being more successful in learning during the lockdown than others with initially equal starting conditions. The data analyses revealed four stable clusters, which are based on different forms of student agency and show potentials and opportunities to support students from educationally disadvantaged backgrounds individually—in homeschooling and in face-to-face teaching.

## Einleitung

Die Corona-Pandemie legt in großer Deutlichkeit Schwachstellen des deutschen Bildungssystems offen, die bereits vor der COVID-19-Pandemie in unterschiedlichen empirischen Befunden expliziert wurden, bislang aber weitgehend unbearbeitet blieben (Sliwka und Klopsch 2020; Bremm und Racherbäumer 2020). So zeigten u. a. die PISA-Studien auf, dass gerade in Deutschland die Differenz zwischen erreichten Kompetenzen von Kindern aus sozioökonomisch stärkeren und schwächeren Elternhäusern so groß ist wie in keinem anderen Land (OECD 2019). Auch wurde deutlich, dass in Deutschland 30 % der Schüler:innen als resilient bezeichnet werden können, d. h. sie liegen aufgrund ihres sozialen, kulturellen und wirtschaftlichen Status’ im untersten Quartil, sind aber bei der Leistungsverteilung im Ländervergleich (nach Berücksichtigung des sozioökonomischen Status) im obersten Quartil zu verorten. Dies bedeutet, dass manche Schüler:innen ihr Potenzial voll ausschöpfen können, wenngleich sie zunächst deutlich ungünstigere Herkunftsbedingungen mitbringen als andere (Schleicher 2018, S. 51). Rohlfs (2011, 2013) konnte zeigen, dass auch an Schulen in benachteiligter Lage vorwiegend günstige Einstellungen der Schule und dem Lernen gegenüber zu finden sind. Ein guter Schulabschluss ist der überwiegenden Mehrheit der Schüler:innen wichtig. Interessanterweise ist diese Zielorientierung bei Jugendlichen mit Migrationshintergrund und bei Mädchen im Allgemeinen oftmals mit einer bemerkenswerten Lernfreude gekoppelt. Gerade aber Jugendliche mit Migrationshintergrund können diese zunächst günstigen Voraussetzungen seltener in Leistung und Schulerfolg umsetzen (Einstellungs-Leistungs-Paradox). Das Wirkungsgefüge zwischen Herkunftsbedingungen, Einstellungen und Leistung im System Schule ist komplex.

Die vorliegende Studie nimmt vor diesem Hintergrund diejenigen Schüler:innen in den Blick, die an Schulen in herausfordernden Lagen unterrichtet werden, und fragt nach deren Einstellungen, Haltungen und Lernerfahrungen während des Lockdowns. Das häusliche Umfeld wird dabei als Lernumgebung in den Blick genommen – nicht als außergewöhnliche und einmalige Lernsituation während der Pandemie, sondern auch unter der Prämisse, dass Lernen immer innerhalb eines Ökosystems stattfindet, welches das häusliche Leben miteinschließt (Bronfenbrenner 1981).

Der Beitrag stellt zunächst entsprechende theoretische Anknüpfungspunkte sowie den aktuellen Stand der Forschung vor (Abschnitt 2), expliziert die Methoden der eigenen Untersuchung (Abschnitt 3) und deren zentrale Befunde (Abschnitt 4), bevor eine Diskussion (Abschnitt 5) und ein Fazit (Abschnitt 6) die Argumentation beschließen.

## Theoretische Rahmung und Stand der Forschung: Lernen in bildungsfernen Familien vor und während der Pandemie

Distanzlernen, das häufig umgangssprachlich auch als Homeschooling bezeichnet wird, ist in Bezug auf das Lernen im deutschen Schulsystem ein vergleichsweise junges Forschungsfeld, das erst zu Beginn der Pandemie an Bedeutung gewann. Viele empirische Studien bauen deshalb auf Modellen auf, die entweder Lernprozesse im häuslichen Umfeld aus der Perspektive der Hausaufgabenforschung betrachten (Huber und Helm 2020) oder das Angebots-Nutzungsmodell für das Homeschooling adaptieren (z. B. Züchner und Jäckel 2021). Helmkes (2015) Grundidee, dass Unterricht stets nur ein Angebot der Lehrenden an die Lernenden sein kann und es von vielfältigen Faktoren – wie etwa der Prozessqualität des Unterrichts, dem individuellen Lernpotenzial der Lernenden, den Kompetenzen und dem Engagement der Lehrperson und weiterer familiärer Merkmale – abhängt, inwiefern dieses Angebot tatsächlich angenommen wird, findet auch für das Distanzlernen seine Bestätigung – jedoch auf anderen Ebenen und im Kontext veränderter Modi. Denn jegliche Form des Lernens, egal ob formal, non-formal oder informell eingebettet, wird durch Kommunikation und Kooperation in direkter Interaktion angeregt. Die Lernsituation während der Pandemie hat dies grundlegend verändert. Während des Lockdowns fand Lernen auf Distanz statt, Lernende und Lehrende waren herausgefordert, neue Wege zu etablieren, um Lernprozesse anzuregen und zu unterstützen bzw. Unterstützung zu erhalten. Sie befanden sich dabei in einer unsicheren (Lern‑)Situation, die auch in Phasen des Wechselunterrichts bzw. der klassenweisen Quarantänen, immer wieder dazu führte, auf Distanz zu lernen. Um die in dieser Zeit stattfindenden Lernprozesse zum Erfolg zu führen, war es erforderlich, die zugrundeliegenden Prozesse zunächst so zu organisieren, dass innerhalb der Familien sichergestellt werden konnte, dass allen Lernenden überhaupt erst einmal die grundsätzliche Möglichkeit zu lernen eröffnet wird. Schule und Familie muss sich dabei noch stärker als zuvor aufeinander einlassen (Buhl und Bonanati 2020). Vorliegende Modelle aus der Beforschung von Hausaufgaben legen nahe, dass der familiäre Kontext starken Einfluss auf die Qualität und den Erfolg von Lernprozessen hat (Hagenauer und Oberwimmer 2019). Zudem beeinflussen die Art der Aufgaben, persönliche Eigenschaften der Schüler:innen sowie die Rolle der Eltern die Motivation, Aufgaben zu erledigen, und diese wiederum das Verhalten beim Erledigen der Hausaufgaben (Trautwein et al. 2006). Der Zusammenhang zwischen Schulerfolg und der eigenen Grundhaltung zum Lernen wird in anderen Studien, bspw. der Befragung des Instituts für Demoskopie Allensbach, die Schüler:innen vor und während der Pandemie befragte, ebenfalls bestätigt (IfD Allensbach 2021).

Die Wirkkette von positiven Einstellungen zur erfolgreichen Leistungserbringung, unter Berücksichtigung des individuellen kognitiven Potenzials, wird in der empirischen Forschung jedoch nicht uneingeschränkt unterstützt. So zeigen unterschiedliche Studien ein Einstellungs-Leistungs-Paradox auf, bei dem eine negative Einstellung zu einem bestimmten Fach dennoch mit hohen Leistungen einher gehen kann (van de Gaer und Adams 2010) oder aber trotz positiver Einstellung vergleichsweise schlechte Leistungen erbracht werden (PISA 2019; Rohlfs 2013).

Zur häuslichen Lernsituation liegen bislang unter der Berücksichtigung soziokultureller Aspekte kaum belastbare empirische Befunde vor (Bräu, Harring und Weyl 2017). Im Allgemeinen gehen Studien davon aus, dass die sozioökonomische Herkunft der Lernenden Lernprozesse wie Lernerfolge beeinflusst (Huber und Helm 2020). Erste Untersuchungen weisen darauf hin, dass dies „insbesondere auf […] fehlenden Fähigkeiten zum selbstgesteuerten Lernen und zur Selbstorganisation des Tagesablaufs“ (Huber und Helm 2020) und damit auf nur wenig ausgeprägten Lernstrategien beruht. Das nur eingeschränkte Vorhandensein solcher Schlüsselkompetenzen wird bei sozial benachteiligten Lernenden auch im Zusammenhang mit Selbstmanagement angenommen (Artelt, Naumann und Schneider 2010): Es wird gemeinhin vermutet, dass erfolgreiche Lernprozesse nur dann erfolgen können, wenn die Schüler:innen selbst dazu in die Lage versetzt werden, eigenständig an ihren Lernprozessen zu arbeiten: „Nur ein Lernen, das auf der Fähigkeit zu einer eigenständigen Organisation der Lernprozesse aufbaut, ist krisensicher.“ (Hoffmann 2020, S. S.100).

Die eigenständige Organisation der Lernprozesse hängt dabei einerseits von den Einstellungen und der Motivation der Lernenden ab. Andererseits wird sie bedingt von den Emotionen, die innerhalb der Lernsituationen auftreten. Dies umfasst nicht nur die Ängstlichkeit oder Zuversicht, Lernprozesse verfolgen zu können, sondern auch bspw. die Stimmung im Elternhaus. Anschließend an Forschung zu häuslichen Lernprozessen kann davon ausgegangen werden, dass für Lernende die Ansprechbarkeit, die Strukturierung und die Kontrolle durch Eltern (Dumont et al. 2014) – und im Distanzlernen auch durch Lehrkräfte – wichtig sind, wenn sie selbstverantwortlich arbeiten. Dies trifft insbesondere für Lernende zu, denen das vergleichsweise offene häusliche Lernszenario weniger entspricht (Fischer et al. 2020). Häufig wird dies Schüler:innen zugeschrieben, die eher leistungsschwach sind und die erforderlichen Kompetenzen zum eigenständigen Lernen wenig trainiert haben (Huber und Helm 2020). Selbstreguliertes Lernen wird so zu einem wichtigen Ziel individueller Förderung (Klieme und Warwas 2011, S. S.813), da gerade im Distanzlernen nur dann individuelle Fördermaßnahmen erfolgreich sein können, wenn die Schüler:innen ihr Lernen zumindest grundständig selbst organisieren können (Hattie und Zierer 2018).

Die im Beitrag referierte eigene empirische Studie adressiert die Fähigkeit, eigene Lernprozesse zu steuern, in Anlehnung an das theoretische Konstrukt der Student Agency. Student Agency impliziert, dass sich die Lernenden als aktive Akteur:innen ihres Lernens wahrnehmen. Sie handeln selbstbestimmt, gestalten ihre eigene (Lern‑)Umwelt und entscheiden verantwortungsvoll (OECD 2020; Sliwka und Klopsch 2022). Unterstützt werden die Lernenden in ihrer Agency durch die Co-Agency ihrer Lehrkräfte, anderen Lernenden und ihrer Eltern (Klopsch und Sliwka 2022).

Generell – während und vor der Pandemie – zeigt sich, dass den Eltern aus allen Bildungsschichten die hohe Bedeutung eines erfolgreichen Schulabschlusses bewusst ist. Ebenso bestätigen Eltern gleichermaßen, dass sie ihre Kinder aktiv zum Lernen animieren (IfD Allensbach 2021). Eltern, die einen mittleren oder einfachen Bildungsabschluss besitzen, unterstützen die Lernprozesse der Kinder in der Folge allerdings weniger als diejenigen mit Abitur oder Hochschulabschluss. Den Eltern unterschiedlicher Bildungsmilieus gemein wiederum ist, dass jüngere Kinder tendenziell mehr unterstützt werden als ältere (ebd.).

Die eigenen Fördermöglichkeiten schätzen Eltern mit niedrigem Bildungsabschluss sowie Alleinerziehende deutlich schlechter ein als die Eltern aus höheren Bildungsschichten (IfD Allensbach 2021). In diesem Zusammenhang zeigte sich, dass die letztgenannten Eltern während des Lockdowns deutlich zufriedener mit dem schulischen Lernangebot waren als die Eltern mit niedrigem Bildungsabschluss (Institut der deutschen Wirtschaft Köln 2021). Bildungsferne Eltern zeigen generell ein höheres Maß an Ängsten im Zusammenhang mit der Gesundheit und der schulischen Entwicklung der Kinder (Vodafone Stiftung 2020).

Insgesamt fällt auf, dass die referierten Studien fast ausschließlich Eltern oder Schüler:innen der weiterführenden Schulen in den Blick nehmen. Jüngere Lernende werden oft vernachlässigt – ebenso wie Lernende aus bildungsfernen Milieus, die nur sehr eingeschränkt berücksichtigt werden und oftmals nicht als Zielgruppe zu identifizieren sind. In dieser Forschungslücke verortet sich vorliegende Studie, indem sie Schüler:innen von Grundschulen „in benachteiligten Lagen“ in den Fokus rückt.

Die den bildungsöffentlichen Diskurs prägende defizitorientierte Beschreibung spezifischer schulischer Rahmenbedingungen als „benachteiligte Lage“ (Beierle et al. 2019), oftmals auch bezeichnet als „herausfordernde Lage“ (Berkemeyer et al. 2017) oder „sozialräumlich deprivierte Lage“ (Bremm et al. 2016) ist für den *pädagogischen* Diskurs dabei zunächst eine unzureichende Begrifflichkeit. Sie verweist aus sozioökonomischer Perspektive lediglich darauf, dass sich in den Lebenswelten der davon Betroffenen „das Phänomen der wohnräumlichen Segregation besonders deutlich zeigt und für dessen Bewohnerinnen und Bewohner Prozesse der Exklusion und der verringerten Teilhabe“ (Fölker et al. 2015, S. 9) zum Lebensalltag gehören. Aus einer sozialwissenschaftlichen Perspektive werden Schulen in „herausfordernder Lage“ häufig mit zusätzlichen Beschreibungen wie der Bildungsferne oder dem Verweis auf einen hohen Anteil von Schüler:innen mit Migrationshintergrund gekennzeichnet (Berkemeyer et al. 2017). Beide Perspektiven tragen dazu bei, gesellschaftsanalytisch Kontexte von Schulsystemen zu beschreiben. Eine unmittelbare pädagogisch angemessene Handlungsweise lässt sich daraus nicht ableiten, da ein Befund wie der Migrationshintergrund zur pädagogischen Diagnose nicht beiträgt, weil er „nur“ sozialwissenschaftlich eine bestimmte Gruppe beschreibt (Hradil 2005; Emmerich 2016). In diesem Bedeutungsverständnis wird in vorliegendem Beitrag der Begriff der „benachteiligten Lage“ oder auch des „bildungsfernen Milieus“ verwandt. Dies verweist in bildungspolitischer Perspektive darauf, dass die Lernenden weder aus wohlhabenden Familien stammen noch aus Akademikerkreisen, was dazu führt, dass sie weniger durch ihr Elternhaus in ihrer Bildungskarriere unterstützt werden (können) als diejenigen, deren Eltern höher gebildet sind (s. oben; iwd 2021). Die Benachteiligung liegt dabei nicht nur im häuslichen Lernen. Auch aus schulischer Perspektive zeigt sich: Kinder in Deutschland, die deren Herkunftsfamilie einen geringen sozioökonomischen Status aufweisen, fallen im internationalen Vergleich in ihrer Kompetenzentwicklung, bspw. im Lesen, besonders stark zurück (PISA 2019). Fraglich dabei scheint zunächst, ob die Benachteiligung dieser Gruppe durch das System befördert wird, bspw. durch Einstellungen und Haltungen der Lehrkräfte, die an Schulen in benachteiligten Lagen unterrichten (Bremm 2020; Sundsbo 2015). Darüber hinaus erscheint fraglich, ob grundsätzlich die individuelle Ausgangsposition der Lernenden, bspw. ihre Einstellung dem Lernen gegenüber, als in besonderem Maße ungünstig eingeschätzt werden kann. Letzterer Aspekt steht hier im Vordergrund.

## Forschungsfrage und Methoden

Die empirische Untersuchung wurde in einem verschachtelten Mixed-Methods-Design konzipiert. Dabei wurden qualitative und quantitative Elemente innerhalb einer schriftlichen Befragung kombiniert. Vorliegender Beitrag referiert die Befunde des quantitativen Teils. Die zugrundeliegende Forschungsfrage lautet:

Welche schulbezogenen Einstellungen zeigen Kinder aus bildungsfernen Milieus im Lockdown während der Corona-Pandemie?

Zur Beantwortung dieser Frage wurde ein Instrumentarium konstruiert, das aus selbst formulierten und adaptierten Items besteht und sich in sechs Teile gliedert:

- *Personenbezogene Daten*: Diese Angaben umfassen neben der Klassenstufe, das Geschlecht und die Frage, ob beide Elternteile zu Hause wohnen und ob im häuslichen Umfeld Deutsch gesprochen wird. Die Itemformulierung wurde dabei an die PISA Studien angelehnt (Walter und Taskinen 2007, S. 345).
- *Lernen zu Hause im Lockdown: *Die Beschreibung des Lernens zu Hause umfasst Items zu Emotionen bezüglich des Lernens sowie die allgemeine Gestaltung des Lernprozesses. Unterschiedliche Items wurden aus bereits existierenden Fragebögen übernommen, bspw. aus „Schule zu Hause in Deutschland“ (Heller und Zügel 2020).

Beispielitems:

- Das Lernen in der Schule hat mir gefehlt.
- Ich hatte Probleme, zu Hause einen ruhigen Platz zu finden.
- Ich wusste immer, was ich zu tun habe.

Alle Items ließen sich fünfstufig likert-skaliert einschätzen (trifft gar nicht zu – trifft eher nicht zu – trifft eher zu – trifft voll zu).

c. *Persönliche Bedeutung von Corona.* Die persönliche Einschätzung der Corona-Pandemie und deren Auswirkungen auf das Wohlbefinden stehen hier im Mittelpunkt. Angelehnt an bereits durchgeführte Studien, innerhalb derer Erwachsene befragt wurden (Andresen et al. 2020) sind hier bspw. die folgenden Items zu finden, die ebenfalls fünfstufig skaliert wurden:
  Ich habe Angst, dass aus meiner Familie jemand krank wird.
  Im Lockdown gab es häufig Streit mit meinen Eltern.
  Im Lockdown gab es häufig Streit mit meinen Geschwistern.
d. *Ausblick auf die Zeit nach der Pandemie.* In dieser Kategorie wurden die Schüler:innen gebeten, positive Dinge in zwei Perspektiven aufzuzeigen, die sie mit der Zeit nach der Pandemie verbinden. Dazu wurden die beiden folgenden offenen Fragen formuliert:
  Gibt es Dinge, die du an der Lockdown-Zeit toll findest und die du genauso weitermachen würdest, wenn Corona vorbei ist?
  Freust du dich auf Dinge, die sich endlich wieder ändern, wenn Corona vorbei ist?
e. *Einschätzungen zur eigenen Person*. Die Einschätzungen der eigenen Person lassen sich dem Konstrukt der Student Agency zuordnen. Hier wurden Items einer Befragung der American Research Association (Zeiser et al. 2018) ausgewählt, die ebenfalls fünfstufig eingeschätzt werden konnten. Beispielitems sind:
  Es gibt Dinge, die ich nicht lernen kann.
  Rückschläge entmutigen mich nicht. Ich gebe nicht so schnell auf.
  Ich bin fleißig.
f. *Einschätzung der eigenen schulischen Leistungen*. In diesem Bereich geben die Schüler:innen darüber Auskunft, inwieweit sie mit ihrer eigenen Leistung zufrieden sind und wie sie diese im Vergleich mit den anderen Kindern der Lerngruppe einschätzen.

Befragt wurden insgesamt *N* = 207 Kinder an vier Grundschulen im Land Berlin, in dem die Grundschule die Klassen 1 bis 6 umfasst. Die Stichprobenziehung erfolgte über eine Auswahl sämtlicher Grundschulen im Land Berlin, die durch eine „benachteiligte Lage“ gekennzeichnet werden können. Innerhalb dieser Schulen wurde dann eine Fokussierung auf die Klassen 3 bis 6 vorgenommen. Die Befragung fand während des pandemiebedingten Lockdowns im Frühjahr 2021 statt. 27,5 % der Schüler:innen besuchten zum Zeitpunkt der Erhebungen die dritte, 24,2 % die vierte, 19,8 % die fünfte und 26,6 % die sechste Jahrgangsstufe. 45 % Prozent waren weiblichen und 55 % männlichen Geschlechts, 19,3 % der Mädchen und Jungen wiesen einen Migrationshintergrund^1^ auf. 51,2 % sprachen zu Hause vorwiegend Deutsch, und 15,9 % sind Kinder von Alleinerziehenden.

Der durch den Fragebogen generierte und aufbereitete Datensatz wurde zunächst uni- und bivariat ausgewertet. Durch Analysen der Fragebogenteile (b) „Lernen zu Hause im Lockdown“ und (c) „Persönliche Bedeutung von Corona“ konnten sechs unterschiedliche Skalen gebildet werden: Lernschwierigkeiten (α = 0,612), Angst vor Noten und Abschlüssen (α = 0,712), Angst um die Gesundheit (α = 0,737), Lernorganisation zu Hause (α = 0,703), das Vermissen der Schule (α = 0,700) und die Stimmung zu Hause (α = 0,704).

Der Itembereich zur Student Agency (e) „Einschätzungen zur eigenen Person“ wurde einer explorativen Faktoren- und Clusteranalyse unterzogen, mit deren Hilfe schließlich Schüler:innentypen charakterisiert werden konnten. Das Kaiser-Meyer-Olkin-Kriterium lag bei 0,747 und der Bartlett-Test fiel hochsignifikant aus (*p* < 0,001), wodurch eine ausreichend hohe Korrelation zwischen den Items indiziert wird, um eine Hauptkomponentenanalyse durchzuführen. Nur Faktoren mit Eigenwerten ≥ 1 wurden in Betracht gezogen (Guttman 1954; Kaiser 1960). Eine Überprüfung des Kaiser-Kriteriums und Scree-Plots rechtfertigte die Extraktion von vier Faktoren, jeweils mit Eigenwerten ≥ 1, die eine Gesamtvarianz von 55,7 % aufklären. Unter den Lösungen lieferte die Varimax-rotierte Vierfaktor-Lösung die Variante, die am besten zu interpretieren war, da sämtliche Items nur auf jeweils einen der vier Faktoren hohe Ladungen ergaben. Tab. 1 zeigt die rotierte Komponentenmatrix.

**Tab. 1:** Rotierte Komponentenmatrix

	Komponente			
	1	2	3	4
*Ich bin zuversichtlich, dass ich bei verschiedenen Dingen gute Ergebnisse erzielen kann*	*–*	*0,680*	*–*	*–*
*Selbst wenn die Dinge schwierig sind, kann ich gute Ergebnisse bringen*	*–*	*0,805*	*–*	*–*
*Wenn ich vor schwierigen Aufgaben stehe, bin ich sicher, dass ich sie bewältigen kann*	*–*	*0,727*	*–*	*–*
*Im Vergleich zu anderen Menschen kann ich die meisten Aufgaben sehr gut erledigen*	*–*	*0,558*	*–*	*–*
Es gibt Dinge, die ich nicht lernen kann	–	–	0,577	–
Wenn ich in einem Fach nicht von Natur aus begabt bin, werde ich darin nie gut abschneiden	–	–	0,748	–
Ich setze mir oft ein Ziel, entscheide mich aber später dafür, ein anderes Ziel zu verfolgen	–	–	0,675	–
*Ich beende, was ich begonnen habe*	*–*	*–*	*–*	*0,669*
*Ich bin fleißig*	*–*	*–*	*–*	*0,541*
*Rückschläge entmutigen mich nicht. Ich gebe nicht so leicht auf*	*–*	*–*	*–*	*0,635*
Ein wichtiger Grund, warum ich meine Schulaufgaben mache, ist dass ich gerne neue Dinge lerne	0,681	–	–	–
Ich nehme mir Zeit, um meine Hausaufgaben zu machen und zu lernen	0,706	–	–	–
Ich gehe gerne in die Schule	0,747	–	–	–
Das Lernen in der Schule bereitet mir Freude	0,741	–	–	–

Alle Konstrukte ließen sich durch eine explorative Faktorenanalyse bestätigen, und in der Hauptkomponentenanalyse wurden entsprechend vier Faktoren extrahiert:

1. Selbstwirksamkeit
2. Dynamisches Selbstkonzept
3. Durchhaltevermögen
4. Lernfreude

Im Anschluss an die Faktorenanalyse wurde eine Clusteranalyse durchgeführt, die vier stabile Cluster für die Lernenden ergab. Die unterschiedlichen Mittelwerte der Ausprägungen aller vier Faktoren zeigten sich bei einer Überprüfung durch eine einfaktorielle ANOVA als hochsignifikant (*p* < 0,001). Es ergaben sich zudem starke Effektstärken nach Cohen (1988) der jeweils einzelnen Faktoren innerhalb der gesamten Clusterlösung (Lernfreude: f = 0,68; Selbstwirksamkeit: f = 0,88; dynamisches Selbstkonzept: f = 1,31, Durchhaltevermögen: f = 0,75). In Post-Hoc-Tests mit Bonferroni-Korrektur fiel auf, dass sich die Lernfreude zwischen Cluster 1 und 2 nicht signifikant (*p* = 0,285) unterschied. Auch im Bereich des Durchhaltevermögens zeigte sich zwischen Cluster 2 und 3 lediglich ein statistisch signifikanter Unterschied (*p* = 0,017) und kein höchst signifikanter Wert, wie er im Rest des Samples jeweils vorliegt.

Limitierend sei darauf hingewiesen, dass die Schulen die Befragung über ihre Online-Tools den Schüler:innen zugänglich machten, diese jedoch auf freiwilliger Basis daran teilnahmen. Es konnte somit keine Vollerhebung unterschiedlicher Schulen erzielt werden. Auch diejenigen Schüler:innen, die keinen digitalen Zugang zu schulischen Materialien hatten, wurden damit automatisch von der Befragung ausgeschlossen. Dies betraf bei allen Schulen weniger als 10 % der Lernenden, was eine Verzerrung der Ergebnisse zur Folge haben kann – allerdings ist fraglich, ob das Vorhandensein digitaler Endgeräte und eines stabilen Internetzugangs in Verbindung mit generellen Einstellungen zu Lernprozessen steht. Diese Schüler:innen waren von den schulischen Lernprozessen nicht ausgeschlossen, sondern erhielten die Materialien und Unterstützung durch die Lehrkräfte auf anderen, analogen Wegen. Eine positive schulbezogene Einstellung ist bei diesen Lernenden nicht ausgeschlossen. So ist durchaus Vorsicht bei der Interpretation der Ergebnisse geboten, eine grundsätzliche Einschränkung der Aussagekraft der vorliegenden Befunde aber liegt nicht vor.

## Ergebnisse

Die Datenanalysen zeigen, dass die befragten Schüler:innen an Schulen in benachteiligter Lage insgesamt über eine positive Einstellung zum individuellen Lernen verfügen (Zustimmung 76 %). Die Stimmung im Elternhaus wird als gut bezeichnet (82 %), wenngleich ihnen die Schule als Lernort im Lockdown fehlt (73 %).

Statistisch signifikante Unterschiede zeigen sich, wenn man die jüngsten befragten Lernenden (3. Klasse) mit den Ältesten (6. Klasse) kontrastiert. Jüngeren Schüler:innen bereitet das Lernen in der Schule mehr Freude (M = 3,25 vs. M = 2,91). Sie nehmen sich mehr Zeit für Hausaufgaben (M = 3,40 vs. M = 2,89) und machen die Schulaufgaben eher, um neue Dinge zu lernen (M = 3,19 vs. M = 2,78). Zudem schildern die Jüngeren häufiger als die Älteren, dass sie die Schule (M = 3,02 vs. M = 2,56) und das Lernen dort (M = 2,93 vs. M = 2,51) vermissen.

Ältere Schüler:innen berichten eher von Problemen, einen ruhigen Lernplatz zu Hause zu finden (M = 3,25 vs. M = 2,67), und davon, beim Lernen zu Hause häufiger gestört zu werden (M = 3,05 vs. M = 2,59). Die älteren Lernenden erhalten mehr Unterstützung durch die Lehrkräfte beim Lernen zu Hause (M = 3,13 vs. M = 2,60), allerdings ist es für sie komplizierter, die Aufgaben von der Schule zu bekommen (M = 3,02 vs. M = 2,65). Dennoch geben sie eher an, zu Hause besser lernen zu können als in der Schule (M = 2,13 vs. M = 1,88).

Interessant erschien in diesem Zusammenhang die Fragestellung nach der Student Agency (OECD 2020), d. h. die Frage, inwiefern die Schüler:innen ihre eigene Handlungsfähigkeit und die damit verbundene Möglichkeit, Lernprozesse positiv beeinflussen zu können, wahrnehmen. In der vorliegenden Studie umfasst die Student Agency vier unterschiedliche Konstrukte, die einerseits empirisch durch die Faktorenanalyse abgesichert wurden, andererseits auch theoretisch an bereits vorhandene Skalen unterschiedlicher Studien anknüpften (Zeiser et al. 2018; Duckworth und Quinn 2009; Consortium on Chicago School Research 2009). Die einzelnen Konstrukte sind (a) die Selbstwirksamkeit, (b) das dynamische Selbstkonzept, (c) die Lernfreude sowie (d) das Durchhaltevermögen bei Lernprozessen. Für die befragten Lernenden lassen sich bezüglich der vier Kategorien ebenfalls vier Cluster ausmachen, die sich statistisch signifikant voneinander unterscheiden:

- Cluster 1: Ausgeprägtes Durchhaltevermögen
- Cluster 2: Statisches Selbstkonzept
- Cluster 3: Dynamisches Selbstkonzept
- Cluster 4: Geringe Lernfreude

Das erste Cluster (37 % aller Befragten) umfasst jeweils die meisten Schüler:innen aller Klassenstufen. Hier finden sich 40 % der Drittklässler:innen, 37 % der Viertklässler:innen, 30 % der Fünftklässler:innen und 37 % der Sechstklässler:innen. 52 % der Kinder in diesem Cluster sind Jungen, 48 % sind Mädchen. Inhaltlich ergab sich, dass hier das Durchhaltevermögen überaus stark ausgeprägt ist. Dieses zeigt sich beispielsweise darin, dass sich die Lernenden als sehr fleißig wahrnehmen und berichten, Lernaufgaben zu beenden, die sie begonnen haben. Zusätzlich kennzeichnet diese Subgruppe eine deutliche Lernfreude, während das dynamische Selbstkonzept im Vergleich eher gering und die Selbstwirksamkeit schwächer vorhanden sind – allerdings nur vergleichsweise, denn insgesamt sind sie immer noch bemerkenswert stark ausgeprägt, was bereits einen relevanten Befund indiziert. Mit Blick auf das zentrale Merkmal dieser Gruppe kann Cluster 1 als „*Ausgeprägtes Durchhaltevermögen*“ bezeichnet werden.

Im zweiten Cluster (24 % aller Befragten) sind 56 % der Kinder männlich und 44 % weiblich. Das Cluster umfasst 35 % aller Kinder aus der 3. Klasse, 28 % der Viertklässler:innen und jeweils 19 % der Schüler:innen aus Klasse 5 und 6. Es besitzt somit die geringste Anzahl an Fünftklässler:innen aller Cluster. Inhaltlich zeichnet sich das Cluster dadurch aus, dass die Selbstwirksamkeit, die Lernfreude und das Durchhaltevermögen zwar stark ausgeprägt sind, das dynamische Selbstkonzept hingegen aber fast nicht vorhanden ist. Dies bedeutet, dass die Lernenden ein eher statisches Konzept ihrer individuellen Fähigkeiten aufweisen. Das zeigt sich beispielsweise in einer deutlichen Verknüpfung von Lernerfolg mit notwendiger Begabung und in der Annahme, dass es Dinge gibt, die sie selbst nicht lernen können. Die Schüler:innen in Cluster 2 können somit als Gruppe mit „*Statischem Selbstkonzept*“ bezeichnet werden.

Das dritte Cluster (18 % aller Befragten) umfasst nur 16 % aller Jungen. Es ist das einzige Cluster, das mehr Mädchen (53 %) als Jungen (47 %) in sich vereint. Aufgeteilt nach Klassenstufen zeigt sich, dass sich hier 16 % der Drittklässler:innen, 20 % der Viertklässler:innen, 25 % der Fünftklässler:innen und 15 % der Sechstklässler:innen verorten. Es ist damit das Cluster, das die wenigsten 6. Klässler:innen umfasst. Das Cluster zeichnet sich dadurch aus, dass die Lernenden ausnahmslos ein dynamisches Selbstkonzept aufweisen. Im Gegensatz zum statischen Selbstkonzept herrscht hier die Annahme vor, sich auch ohne spezielle Begabungen, fachliche und überfachliche Kompetenzen aneignen zu können. Zusätzlich sind ihr Durchhaltevermögen und ihre Selbstwirksamkeitserwartung stark ausgeprägt. Lediglich die Lernfreude zeigt sich in etwas schwächerem Maß. Die Schüler:innen des Clusters 3 können somit – wiederum mit Blick auf das herausragende Merkmal – als Gruppe mit „*Dynamischem Selbstkonzept*“ beschrieben werden.

Im vierten Cluster (21 % aller Befragten) befinden sich 9 % aller Drittklässler:innen sowie 15 % aller Viertklässler:innen. Es ist das Cluster mit den wenigsten Kindern aus Klasse 3 und 4. Zusätzlich befinden sich hier 25 % aller Fünftklässler:innen sowie 29 % aller Sechstklässler:innen. Es ist das zweitgrößte Cluster aus Perspektive der Sechstklässler:innen. Dieses Cluster umfasst am wenigsten Mädchen (17 % aller Mädchen). Insgesamt besteht es zu 61 % aus Jungen und zu 39 % aus Mädchen. Während auch in diesem Cluster das Durchhaltevermögen, das knapp die Hälfte aller Beteiligten als individuell-spezifisches Merkmal sieht, und die Selbstwirksamkeit durchaus ausgeprägt sind, ist das dynamische Selbstkonzept nur sehr zurückhaltend vorhanden. Insbesondere aber eine eingeschränkte Lernfreude bzw. wenig ausgeprägte Begeisterung bezüglich schulbezogener Aspekte kennzeichnen diese Subgruppe, die somit mit der Bezeichnung „*Geringe Lernfreude*“ umschrieben werden kann.

Die unterschiedlichen Cluster weisen nicht nur inhaltlich differente Schwerpunkte im Bereich der Student Agency aus. Auch in Bezug auf die entwickelten Skalen im Bereich der Lernschwierigkeiten, der Angst vor Noten und Abschlüssen, der Angst um die Gesundheit, der Lernorganisation, dem Vermissen der Schule sowie der Stimmung zu Hause, können deutliche Unterschiede ausgemacht werden.

Insgesamt zeigte sich, dass die Stimmung zu Hause sehr positiv eingeschätzt wird. Die Schüler:innen mit* dynamischem Selbstkonzept* erleben überhaupt keine schlechte Stimmung – weder zwischen den Eltern und Kindern, noch zwischen den Eltern oder zwischen Geschwistern – und auch bei der Subgruppe mit* statischem Selbstkonzept* oder den Schüler:innen mit* ausgeprägtem Durchhaltevermögen* liegt der Prozentsatz der schlechten Stimmung bei nur 13 %. Lediglich die Befragten im Cluster mit *geringer Lernfreude* schildern zu knapp 40 % eher schlechte bzw. schlechte Stimmung im Elternhaus (vgl. Tab. 1).

Lernschwierigkeiten im Sinne, dass die Schüler:innen Probleme haben, morgens mit ihren Schulaufgaben anzufangen, oder dass sie sich leicht ablenken lassen bzw. keinen ruhigen Platz finden, schildern am häufigsten die Schüler:innen mit *geringer Lernfreude *(69 %). Das Cluster mit* dynamischem Selbstkonzept* (45 %) weisen hier die geringsten Probleme auf.

Die Gruppe mit *geringer Lernfreude *vermisst die Schule zudem mit Abstand am wenigsten (30 %), während die Schüler:innen mit* ausgeprägtem Durchhaltevermögen* und *dynamischem Selbstkonzept* am häufigsten das Vermissen der Schule berichten (über 70 %).

**Abb. 1:**
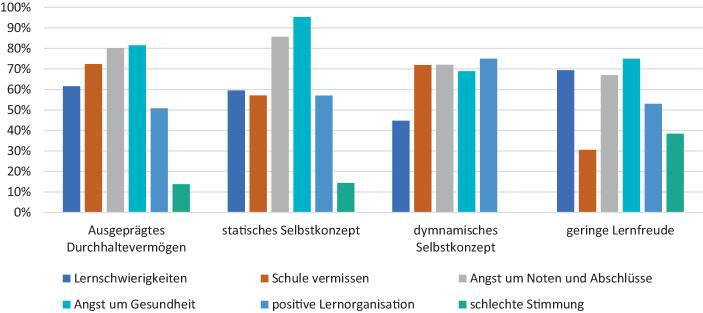
Lernsituation während Corona nach Clustern

Im Bereich der Angst um Noten und Abschlüsse sowie der Angst um die eigene Gesundheit bzw. die von Freunden und Familie fällt auf, dass in allen Clustern, außer in der Subgruppe mit *dynamischem Selbstkonzept* die Ängste am stärksten von allen Skalen (75–95 %) ausgebildet sind. In diesem Cluster steht dagegen eine positive Lernorganisation zu Hause, die auch Elemente der Freude darüber, zu Hause arbeiten zu können, miteinschließt, im Vordergrund (75 % Zustimmung). Ein Aspekt, der von allen anderen Clustern deutlich weniger betont wird (vgl. Abb. 1).

## Diskussion

Die vorliegende empirische Untersuchung gibt Einblick in die persönlichen Einschätzungen von Schüler:innen an Schulen in besonders herausfordernden Lagen. Deren Lebenssituation lässt sich auf den ersten Blick als scheinbar homogen einschätzen und wurde bislang im Zusammenhang mit Bildungserfolg in der Literatur als eher kritisch dargestellt (bspw. OECD 2019). Die referierten Erkenntnisse bestätigen einerseits unterschiedliche Aspekte des vorliegenden empirischen Forschungsstandes, geben aber auch Einblick in Befunde, die so zunächst nicht erwartbar waren, wie im Folgenden dargestellt wird. Zunächst wird hierbei auf Erkenntnisse auf der Einzelitemebene eingegangen, bevor die Ergebnisse der Clusteranalyse diskutiert werden.

So berichten die älteren Schüler:innen, dass sie mehr Unterstützung von den Lehrkräften erhalten, während die Jüngeren mehr von den Eltern unterstützt zu werden scheinen. Ein Befund, der sich im Bereich der bildungsferneren Elternhäuser mit vorliegenden Studien deckt (IfD Allensbach 2021). Die weniger intensive Unterstützung älterer Lernender durch ihre Eltern könnte ein Erklärungsansatz für die weiteren deskriptiven Befunde auf Einzelebene sein, die davon zeugen, dass die älteren Schüler:innen den Zugang zu den Lernaufgaben als komplizierter empfinden als die Jüngeren, die auch darin wahrscheinlich von den Eltern stärker unterstützt werden. Die Älteren werden zu Hause beim Lernen häufiger gestört und haben häufiger Probleme einen ruhigen Lernplatz zu finden. Diese Befunde könnten auch damit zusammenhängen, dass die Lernprozesse der Älteren einen längeren Zeitraum umfassen als die der Jüngeren, wodurch höhere Anforderungen an einen ruhigen Arbeitsplatz gestellt werden, als dies bei einer eher kurzen Bearbeitungsdauer von Aufgaben der Fall ist. Insgesamt scheint die dabei entstehende Unruhe die Älteren aber nicht allzu stark zu beeinträchtigen, da sie davon ausgehen, dass sie zu Hause besser lernen können als in der Schule. Es ist möglich, dass die generell höhere Zufriedenheit mit den häuslichen Lernprozessen auch damit einher geht, dass sie weniger gerne zur Schule gehen als die Jüngeren, die nicht nur die Schule, sondern auch das Lernen dort stärker vermissen.

Überraschend ist der Befund insofern nicht, da bereits mehrere empirische Studien berichten, dass die Motivation, zu lernen und zur Schule zu gehen, im Laufe der Schulzeit deutlich abnimmt (Becker und Staub 2019; Rohlfs 2013). Dem entspricht auch der Befund, dass sich die Schüler:innen mit *geringer Lernfreude *(Cluster 4) hauptsächlich aus den älteren Lernenden zusammensetzen und dass sich in den Clustern mit Schüler:innen, die sich als fleißig wahrnehmen (Cluster 2 und 3), vergleichsweise viele jüngere Lernende und weniger ältere Kinder befinden.

Auffällig ist, dass die positive Lernorganisation einherzugehen scheint mit einem geringerem Maß an Lernschwierigkeiten – beides trifft in den Clustern mit den fleißigen Schüler:innen zu. Diejenigen Schüler:innen, die kaum Störungen beim häuslichen Lernen erleben, berichten vermehrt davon, ihr Lernen selbst gut einteilen zu können und dabei auch Freude zu empfinden. Innerhalb derjenigen Cluster, in denen eigene Lernschwierigkeiten höher eingeschätzt werden, ist die Lernorganisation weniger positiv ausgeprägt. Für die schulische Auseinandersetzung mit Lernprozessen würde dies bedeuten, dass Schüler:innen darin unterstützt werden sollten, eigenes Lernen auch in der häuslichen Umgebung selbständig zu organisieren – auch um womöglich die Freude am Lernen zu steigern.

Ein wichtiger Aspekt innerhalb der Lernprozesse scheint das dynamische Selbstkonzept zu sein. Diejenigen Lernenden, die kein ausgeprägtes dynamisches Selbstkonzept aufweisen, scheinen deutlich mehr Ängste in Bezug auf ihre Noten, ihre Abschlüsse wie auch ihre Gesundheit bzw. die ihrer Freunde und Familie zu haben. Dies zeigt sich besonders deutlich in der Abgrenzung der Fleißigen mit und ohne dynamischem Selbstkonzept. Die Wahrnehmung, Situationen ausgeliefert zu sein und diese nicht aktiv handelnd steuern zu können, scheint sich nicht nur auf die Lernprozesse an sich auszuwirken, sondern das tägliche Leben auch in weiterer Hinsicht zu beeinträchtigen.

Insgesamt zeigt sich, dass ein gutes Drittel (37 %) aller Schüler:innen dem ersten Cluster mit ausgeprägtem Durchhaltevermögen zuzurechnen ist. Die zweitgrößte Gruppe (etwa ein Viertel (24 %)) bildet das Cluster mit *statischem Selbstkonzept*. Das Cluster mit* geringer Lernfreude* macht die zweitkleinste Gruppe mit etwa einem Fünftel (21 %) aller Schüler:innen aus, während sich das Cluster* mit dynamischem Selbstkonzept* lediglich aus 18 % aller Lernenden zusammensetzen. Unter Berücksichtigung der positiven Lerneinstellung und der individuellen Lernausgangslage, auch im Zusammenspiel mit Lernorganisation und Lernfreude, wäre es dringend angeraten, am dynamischen Selbstkonzept der Lernenden zu arbeiten – auch oder gerade unter der Prämisse, Ängste verringern zu können, wodurch Lernprozesse wiederum erleichtert werden.

Überraschend erscheint die positive Stimmung in den Elternhäusern während des Lockdowns. Eine pauschalisierende Einschätzung über schlechte häusliche Lernvoraussetzungen, gerade auch im emotionalen Bereich ist somit unangemessen. Dies bestätigt auch die hohe Lernfreude, die als starker Faktor sowohl bei den Schüler:innen mit ausgeprägtem Durchhaltevermögen als auch in den Clustern mit *statischem* und* dynamischem Selbstkonzept* vorhanden ist. Auch die hohe Selbstwirksamkeit sowie das Durchhaltevermögen, das in allen Clustern deutlich vorhanden ist, weist auf positivere Lernausgangslagen hin, als weithin in der Literatur befürchtet wird.

Eine Unterstützung der schulischen Lernprozesse an Schulen in „benachteiligten Lagen“ könnte demnach besonders im Sinne des kompensatorischen Ansatzes (v. Ackeren 2008) erfolgsversprechend sein, dem zufolge eine Priorisierung sozial-emotionaler Arbeit vor akademischer Leistungsförderung und ein zusätzliches Engagement in der Beziehungsarbeit Wirksamkeit entfalten können. Ausschlaggebend für den Erfolg entsprechender Bemühungen ist es, dass sich nicht nur einzelne Lehrpersonen unabhängig von einander in diesem Kontext engagieren, sondern alle Akteure gemeinsam – gestützt durch die Schulleitung – ein Konzept erarbeiten, das allen Lernenden an der Schule gerecht wird.

Vor dem Hintergrund der in vorliegendem Beitrag referierten Erkenntnisse entsteht die Frage, warum es in Deutschland mit Blick auf den starken Zusammenhang zwischen sozioökonomischer Lage der Lernenden und deren Kompetenzerwerb nicht gelingt, erfolgreichere Lernprozesse bei Schüler:innen aus bildungsfernen Milieus anzuregen. Hierin erkennen wir ein deutliches Forschungsdesiderat und empfehlen zu untersuchen, welche Einstellungen und Haltungen Lehrkräfte an Schulen in herausfordernden Lagen ihren Schüler:innen und deren Lernprozessen und -potenzialen gegenüber zeigen. In diesem Kontext wäre es angesichts des bundesweiten Lehrkräftemangels interessant zu erheben, wie groß die Anzahl von Vertretungslehrkräften, Quereinsteiger:innen bzw. hoch qualifizierter Lehrkräfte an diesen Schulen ist und welche weiteren Unterstützungsmaßnahmen für die Schüler:innen an diesen Schulen bereitgestellt werden müssten, um das positive Potenzial der Lernenden stärker auszuschöpfen.

Neben diesen lehrer:innenspezifischen Fragestellungen ergibt sich insbesondere mit Blick auf die in vorliegender Studie untersuchte spezifische Zielgruppe ein erhöhter Forschungsbedarf. So erscheint es von hoher Relevanz, im Rahmen weiterführender Forschung die Rahmenbedingungen und Wirkmechanismen zu untersuchen, die dazu führen, dass sich die vielfach günstigen Einstellungen von Kindern und Jugendlichen an Schulen in benachteiligter Lage oftmals nicht in Lernerfolg abbilden. Dieses Einstellungs-Leistungs-Paradoxon betrifft insbesondere Schüler:innen mit Migrationshintergrund. Gewinnbringend wären hier Mixed-Methods-Designs, die vertiefend diesen paradoxen Zusammenhang aufklären könnten. Die Gründe für eine günstige Einstellung zur Schule trotz herausfordernder Umstände wären hier ebenso interessant wie die Ursachen für den damit verbundenen schulischen Misserfolg.

## Fazit

Lernende an Schulen in herausfordernden Lagen lassen sich nicht pauschalisierend zu den „Bildungsverlierern“ zählen, die den eigenen Lernprozessen neutral bis negativ gegenüberstehen. Die Lernausgangslage, d. h. die Einstellungen und Haltungen der Lernenden gegenüber ihren Lernprozessen, zeigt sich im vorliegenden Sample positiver, als dies vielfach vermutet wird, und dies auch im Lockdown während der Corona-Pandemie. Die deutliche Mehrheit der befragten Schüler:innen zeigen günstige schulbezogene Einstellungen, lediglich ein Fünftel der Befragten lassen sich bspw. dem Cluster mit geringer Lernfreude zuordnen. Im Grunde ergeben sich hier – zumindest aus dieser Perspektive – ideale Voraussetzungen für schulisches Lernen. Dennoch scheint es eine Herausforderung für das deutsche Schulsystem zu sein, das vorhandene Potenzial von Lernenden so zu nutzen, dass sie erfolgreich schulische Lernprozesse durchlaufen. Wenn das Distanzlernen im Lockdown also insbesondere Kinder und Jugendliche aus bildungsfernen Milieus benachteiligt und sich die Schere zwischen Privilegierten und Zurückgelassenen, die in Deutschland ohnehin stark geöffnet ist, durch die COVID-19-Pandemie immer deutlicher weitet, liegt das aus Sicht der befragten Schüler:innen an Schulen in herausfordernder Lage nicht in ihren schulbezogenen Einstellungen begründet, nach deren Ausprägung der vorliegende Beitrag fragt. Die generierten Befunde hinterfragen pauschalisierende und defizitorientierte Zuschreibungen bezüglich dieser Zielgruppe. Auch die Atmosphäre in den Familien im Lockdown erscheint weniger negativ als vor dem Hintergrund des fachöffentlichen Diskurses zu erwarten.

Der Umkehrschluss darf nun gewiss nicht lauten, dass hier keine Benachteiligungen und Probleme vorliegen. Das wäre eine verkürzte und riskante Interpretation der vorliegenden Ergebnisse. Vielmehr gilt es dezidiert die Mechanismen zu untersuchen, die die Wirkung günstiger Einstellungen vielfach einschränken, und entsprechende Handlungsmöglichkeiten zu entwickeln. Dafür erscheinen die Ergebnisse zur Student Agency relevant und lenken den Blick auf die Frage, wie stark sich die Lernenden als aktive Akteur:innen ihres Lernens wahrnehmen und wie sehr sie sich als selbstbestimmt handelnd und selbständig entscheidend in der Organisation ihrer Lernprozesse und Gestaltung ihrer eigenen (Lern‑)Umwelt erleben.

Einen Ansatzpunkt in diesem Kontext könnte das dynamische Selbstkonzept liefern – das implizieren die Befunde der im Beitrag referierten empirischen Studie. Dieses liegt nicht nur im Zusammenhang mit einer positiven Lernorganisation und geringen Lernschwierigkeiten vor, sondern ist auch in dem Cluster stark ausgeprägt vorhanden, das die weniger ängstlichen Schüler:innen in sich vereint. Das Cluster stellt jedoch die kleinste der vier Subgruppen, wodurch ein deutlicher Handlungsbedarf indiziert wird. Um das dynamische Selbstkonzept zu steigern scheint es nicht nur angemessen, auf der Ebene der schulischen Lernprozesse den jeweils individuellen Lernprozess und die damit verbundenen Potenziale zu thematisieren, sondern auch die häusliche Lernumgebung einzubeziehen – sowohl in Zeiten von Präsenzunterricht als auch im Rahmen digitaler Lernarrangements.

## Footnotes

1 Definiert analog zu PISA (vgl. Walter und Taskinen 2007, S. 345).
